# Impact of Visual Input and Kinesiophobia on Postural Control and Quality of Life in Older Adults During One-Leg Standing Tasks

**DOI:** 10.3390/vision9010024

**Published:** 2025-03-20

**Authors:** Paul S. Sung, Dongchul Lee

**Affiliations:** 1 School of Nursing and Health Professions, Department of Physical Therapy, Indiana Wesleyan University, Marion, IN 46953, USA; 2 Neurostim Insight, Santa Clarita, CA 91390, USA; dreamittogether@gmail.com

**Keywords:** vision, postural sway velocity, fear of movement, one-leg standing, quality of life

## Abstract

Visual conditions significantly influence fear of movement (FOM), which is a condition that impairs postural control and quality of life (QOL). This study examined how visual conditions influence sway velocity during repeated one-leg standing tasks and explored the potential relationship between postural control, FOM, and QOL in older adults with and without FOM. Thirty-seven older adults with FOM and 37 controls participated in the study. Postural sway velocity was measured across three repeated trials under visual conditions in both anteroposterior (AP) and mediolateral (ML) directions. The groups demonstrated significant interaction under visual conditions (F = 7.43, *p* = 0.01). In the eyes-closed condition, the FOM group exhibited faster ML sway velocity than the control group, with significant differences across all three trials. There was a significant interaction between sway direction and vision (F = 27.41, *p* = 0.001). In addition, the FOM demonstrated strong negative correlations with several QOL measures on social functioning (r = −0.69, *p* = 0.001) and role limitations due to emotional problems (r = −0.58, *p* = 0.001) in the FOM group. While FOM influenced sway velocity during balance tasks, visual input emerged as a key determinant of postural control. The FOM group demonstrated a heightened reliance on vision, suggesting an increased need for vision-dependent strategies to maintain balance.

## 1. Introduction

Individuals with visual impairment exhibit deficits in both dynamic and static balance, which adversely impact motor control [1]. Recent reviews suggest that balance deteriorates more significantly in older adults with visual impairments, requiring greater reliance on proprioception and the vestibular system to compensate for visual deficits [2,3]. Individuals at high risk of falls possess deficits in vestibular function and the ability to integrate sensory information under challenging conditions, which have been identified as critical contributors to balance impairment [4]. To counter these challenges, those individuals with fear of movement (FOM) often rely more effectively on somatosensory input to adjust for dynamic balance deficits [5,6,7].

Kinesiophobia, characterized by FOM due to pain or injury, presents a significant barrier to functional recovery and physical activity in individuals with chronic musculoskeletal conditions [6,8]. This fear often leads to avoidance behaviors, reduced mobility, and impaired balance control, which can negatively impact physical function and serve as a predictor of future falls [9]. However, some studies report conflicting findings, suggesting that visual conditions may not consistently threaten postural balance control, particularly in older adults [10]. Given the critical role of vision in maintaining balance, particularly in eyes-closed conditions, the processing of visual stimuli may significantly influence static balance control [11,12]. Understanding these interactions is essential for developing targeted strategies that address sensory-motor factors in individuals with FOM.

A previous report indicated that visual input plays a crucial role in postural control for older adults, as maintaining balance relies on the integration of visual, vestibular, and proprioceptive systems [3]. Despite its significant impact on mobility and independence, the relationship between FOM and postural control under visually challenging conditions remains poorly understood. When visual feedback is unavailable or reduced (e.g., during eyes-closed conditions), individuals with FOM may exhibit heightened instability and increased anxiety about falling, which further amplifies their difficulty in modulating postural sway.

The ability of older adults with FOM to adjust their postural control in response to sway velocity changes during one-leg standing tasks has not been thoroughly examined. A recent study reported that the relationship between postural sway and FOM can hinder recovery from low back pain [13]. However, older adults demonstrated reduced accuracy in mental navigation tasks and exhibited increased anteroposterior (AP) and mediolateral (ML) sway velocity in a closed stance [14]. Their results indicated that older adults showed higher AP sway velocity when standing in a more challenging stance. However, there is limited understanding of how visual condition influences postural control in older adults with FOM. In addition, age-related declines in sensory and motor functions further increase reliance on visual feedback for stability [15]. Previous systematic reviews have emphasized the need to examine sensory-motor contributions to balance impairments in older adults [3,16].

Older adults generally exhibit impaired postural control, characterized by increased postural sway, altered sensory reliance, and overdependence on vision for balance maintenance [17,18]. This pattern supports the hypothesis that FOM negatively impacts standing balance. FOM has been associated with reduced motor control efficiency and heightened postural instability, particularly in eyes-closed conditions. Individuals with high FOM frequently adopt a stiffening strategy, display decreased postural adaptability, and experience increased sway in situations requiring sensory reweighting [19,20]. These balance deficits likely result from heightened fear-avoidance behaviors, which contribute to altered motor planning and maladaptive postural adjustments [21].

The Tampa Scale of Kinesiophobia (TSK) is widely used to assess FOM and its impact on postural control in older adults [22,23]. FOM, characterized by an excessive fear of physical activity due to anticipated pain or injury, is a major concern, particularly for those with balance impairments [7]. In addition, the interaction between somatosensory reliance and psychological factors highlights the critical role of visual conditions in modulating FOM. This relationship impacts both postural control and quality of life (QOL), as heightened kinesiophobia leads to reduced physical activity, increased fall risk, and diminished engagement in daily activities [24,25]. In older adults with FOM, specific domains, such as physical functioning and bodily pain, are often disproportionately impacted due to physical limitations and chronic discomfort. Similarly, emotional and social functioning scores may reflect the psychological and social consequences of reduced mobility and increased fall risk [24]. This evidence highlights the critical need to explore the compensatory sway velocity changes and QOL outcomes in older adults with and without FOM. Understanding these interactions is essential for developing targeted interventions aimed at improving functional independence, reducing fall risk, and enhancing the overall QOL in older adults with FOM.

The short-form 36-item questionnaire (SF-36) has been utilized to assess individuals’ QOL across eight health domains [26,27]. The SF-36 provides a reliable and validated framework for assessing the multidimensional effects of FOM on QOL, making it a valuable tool. By identifying the deficits in specific domains, the SF-36 can inform targeted interventions designed to address the unique challenges associated with FOM, such as physical functioning. Effective postural control depends on the integration of sensory inputs and motor outputs, but FOM disrupts this balance, particularly during dual-task performances requiring increased attentional resources [28,29,30]. As sensory input decreases, the attentional demands for maintaining stability rise, contributing to imbalance and increased fall risk. Therefore, older adults with FOM often exhibit altered motor control and compensatory postural adjustments, which affect their functional independence and QOL.

In addition, center of pressure (COP) metrics have been extensively studied as indicators of balance control, particularly in evaluating sway distances during one-leg standing tasks [31,32,33]. The COP-based sway velocity assessments focus on AP and ML sway directions, which are critical for understanding balance control mechanisms. For example, a participant may compensate for the lack of visual input by employing larger or faster postural adjustments if higher velocities are observed in eyes-closed conditions. One-leg standing is a well-established clinical balance test for evaluating postural control in older adults and is crucial for functional mobility [34,35,36]. Maintaining balance is essential for reducing fall-related injuries and performing daily activities. However, the clinical utility of one-leg standing balance assessments, especially when incorporating visual conditions alongside FOM, remains unclear. While some studies highlight their effectiveness in identifying postural control deficits, others question their relevance and note gaps in their clinical application [36,37,38]. Although COP metrics provide valuable insights into balance control, inconsistencies in the literature regarding the applicability of one-leg standing balance tasks underscore the need for further research. Addressing these gaps is essential to translating findings into effective clinical assessments, particularly for older adults with FOM and balance impairments.

The purpose of this study was to investigate (1) the influence of visual conditions on sway velocity during repeated one-leg standing tasks and (2) the relationship between TSK and QOL measures in older adults with and without FOM. We hypothesized that older adults with FOM would exhibit higher sway velocities in the eyes-closed condition. In addition, the QOL scores will be different between groups with and without FOM.

## 2. Methods

### 2.1. Participants

Subjects were sourced from the community via targeted advertising. Individuals meeting the study’s inclusion criteria were comprehensively briefed about the research objectives and procedures, and informed consent was duly obtained. The study was conducted in strict compliance with the guidelines set forth by the Institutional Review Board (IRB #1653.21). Eligibility for participation was determined based on the following criteria: (1) age between 50 and 90 years; (2) right-limb dominant; (3) absence of severe pathologies, such as nerve root compromise, at the time of data collection; and (4) no existing conditions that would preclude one-leg standing. The exclusion criteria included the following: (1) a diagnosed psychological disorder that could potentially disrupt the study protocol; (2) manifestation of neurological symptoms, including sensory deficits or motor paralysis; and/or (3) pregnancy. In recruiting the control group, considerations were made for anthropometric variations related to age and body mass index (BMI). The study focused on the dominant sides of both the upper and lower limbs, as previous research has identified limb dominance as a potential confounding factor in postural control and motor performance [39,40]. To ensure a consistent and standardized approach in minimizing limb dominance effects on sway measurements, the right lower limb was designated as the dominant side for all subjects based on their self-reported preference for using the right limb to kick a ball [41,42].

As shown in Table 1, seventy-four older adults participated in the study. Thirty-seven subjects without FOM (22 female and 15 male) and 37 with FOM (20 female and 17 male) participated in the study. There was no significant group difference on gender (χ^2^ = 0.11, *p* = 0.74), age (*t* = 0.23, *p* = 0.41), or BMI (*t* = −0.59, *p* = 0.27). However, the FOM group had significantly higher TSK scores (*t* = −11.78, *p* = 0.001). The SF-36 demonstrated significant group differences except SF36-EF (*t* = 1.59, *p* = 0.06).

### 2.2. Experimental Procedures

Upon arrival at the lab, individual health status questionnaires, including demographic data, were collected. The TSK scale was utilized to assess FOM, or the level of kinesiophobia [22,23]. It was developed to differentiate between non-excessive fear and phobia in patients with chronic musculoskeletal pain, specifically assessing FOM in individuals with persistent pain conditions. In addition, its relevance has been explored in individuals experiencing acute pain [43], as heightened fear-avoidance behaviors in the acute stage may contribute to the transition from acute to chronic pain as well as affect different musculoskeletal regions [44,45]. The TSK is a 17-item self-report questionnaire with a total score ranging from 17 to 68. It utilizes a 4-point Likert scale based on the fear-avoidance model, evaluating fear related to movement, work-related activities, and re-injury [46]. Kinesiophobia is caused by excessive sensitivity to the pain experience among older adults, and it was reported as a central factor in the process of pain developing from acute to chronic stages [47]. In a previous study, the psychocognitive factor that hampers recovery in patients with TSK ≥ 37% or 35% was allocated to the kinesiophobia group [45,48].

The RAND short-form 36-item questionnaire (SF-36) is a widely used tool for assessing individuals’ QOL across eight health domains: physical functioning (PF), role limitations due to physical health (RP), role limitations due to emotional problems (RE), energy and fatigue/vitality (VT), emotional well-being/mental health (MH), social functioning (SF), bodily pain (BP), and general health (GH). Each subscale score is converted to a 0–100 scale, where higher scores indicate a better health-related QOL [26,27]. The SF-36 is a well-validated, reliable, and internally consistent measure for evaluating health status and QOL, particularly in individuals with chronic LBP. It is highly responsive to changes in health status, with a minimal detectable change of only 20 points across all eight domains. This sensitivity makes it an effective tool for tracking QOL outcomes, including physical and emotional health, pain levels, and overall well-being. The SF-36 was utilized to assess QOL as a multidimensional approach for a comprehensive evaluation of the individuals’ daily functioning and emotional well-being, supporting the identification of specific areas of impairment that may benefit from targeted rehabilitation interventions.

**Figure 1:**
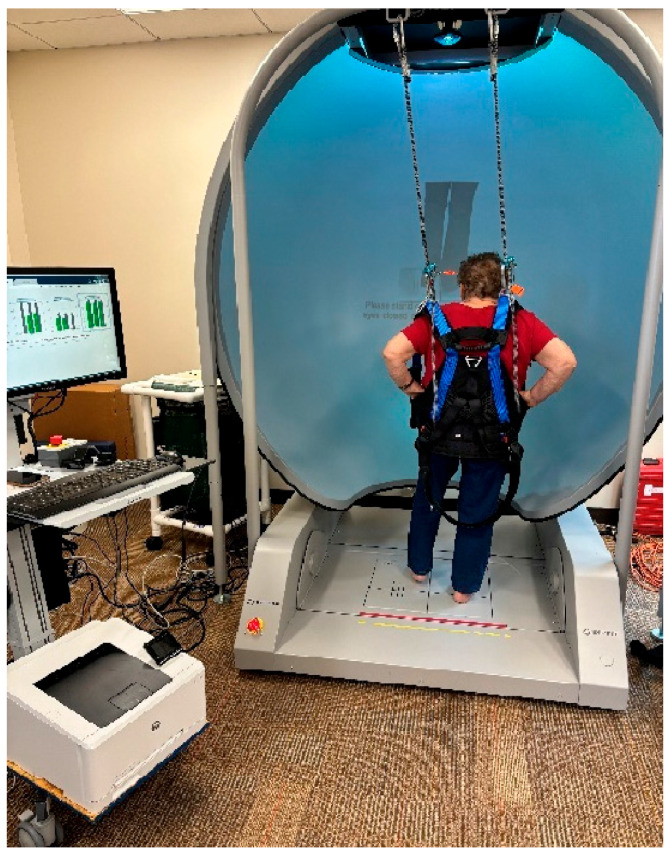
Initial setup for the one-leg standing test using the Bertec^®^ device. A subject was secured in a full-body safety harness and instructed to stand on his/her dominant leg for the duration of each trial following specified visual conditions. Each subject stood barefoot on one leg for 10 s, with the opposite knee flexed at approximately 30° while maintaining the standing leg in a vertical position. A 10 s rest interval was provided between each trial.

Each subject underwent a standardized testing procedure to assess postural control during one-leg standing on the dominant limb. Participants were instructed to remove their footwear and stand barefoot on a pair of Bertec force plates (FP4060-08, 40 cm × 60 cm; Bertec Inc., Columbus, OH, USA), which are considered the gold standard for balance assessment. The medial malleolus was aligned with a horizontal guideline to ensure consistent positioning across trials. Standardized test protocols for one-leg standing tasks were administered, as illustrated in Figure 1. All assessments and examinations were conducted by the same trained examiner to maintain uniformity. The ankle joint was aligned with the transverse axis and the lateral side of the calcaneus. The *y*-axis indicated AP movements on the platform, while side-to-side movements on the support surface occurred along the *x*-axis (ML movements) for the calculation of sway velocities.

A full-body safety harness system, secured to an overhead bar, was worn by subjects to prevent fall injuries [4,6,49,50]. The subjects stood on the Bertec Balance Advantage^®^ system for Computerized Dynamic Posturography with Immersion Virtual Reality (CDP-IVR) with their feet positioned comfortably apart. The CDP-IVR allowed for the measurement of balance performance and the monitoring of postural stability improvements. The one-leg standing test was conducted by the system to assess postural stability under two different visual conditions: eyes open and eyes closed. The order of these conditions was randomized to minimize potential learning effects and bias. Each standardized procedure was demonstrated to the subjects before testing. Initially, subjects were asked to stand steadily on their dominant foot for 10 s on the balance plate with their eyes open. Upon instruction, the subject stood upright on the force plate with the contralateral hip and knee flexed to approximately 30 degrees. Subjects were instructed to place their hands on their hips to eliminate upper-limb contributions to balance, thereby isolating lower-limb and core postural control mechanisms.

The ground reaction force was recorded at a sampling frequency of 1000 Hz. The kinetic data were filtered and normalized based on individual body weight to ensure accurate comparisons. A low-pass Butterworth filter was applied to the COP data to reduce high-frequency noise and smooth sway velocity calculations [51]. The raw force and moment data were pre-processed to eliminate transient fluctuations and artifacts. This normalization ensures that postural sway differences reflect balance control rather than anthropometric variations. The manufacturer calibrated the force plate, and a sensitivity matrix was provided to convert the voltages to forces and torques.

The data were collected from an unloaded platform to determine the zero offset, and changes in balance during one-leg stance balance tasks were recorded. The force plates demonstrated moderate to very high reliability across various postural sway measures [52]. The data included only subjects who successfully completed the full 10 s standing duration as requested in the test protocol. All subjects were able to meet this requirement, ensuring consistency in data collection. Regarding the kinetic data, the COP sway velocities in the ML and AP directions were analyzed. The force plate data were used to calculate COP AP (Mx/Fz) and COP ML (-My/Fz), and only trials with three valid test repetitions per subject were included.

### 2.3. Statistical Analysis

A power analysis was conducted based on the pilot data, and the sample size calculation determined that a minimum of 30 subjects per group would provide 80% power to detect an effect size of 0.4 [53]. The effect sizes were analyzed by partial Eta-squared values (η^2^p) within repeated-measures analysis of variance (ANOVA) squared (small ≥ 0.01, medium ≥ 0.06, large ≥ 0.14), which was used to indicate the mean difference between groups. The independent variables included group classification (with and without FOM), visual condition (eyes open and eyes closed), and three repeated trials to assess sway velocity changes.

An independent *t*-test was utilized to investigate differences in individual characteristics between the groups. A mixed repeated-measures ANOVA was conducted to examine the main and interaction effects on sway velocities when considering visual conditions. Using a by-group factorial experimental design, the general linear model assessed all continuous dependent variables.

In cases where demographic factors showed significant group differences, these variables were included as covariates in the analyses. Controlling these individual characteristics is critical for accurately interpreting dynamic standing balance strategies. Failure to account for such confounding variables could limit the generalizability of the findings. All statistical analyses were performed using SPSS version 28.0 (IBM Corp, Armonk, NY, USA) with a significance level set at 0.05 for all tests.

## 3. Results

The results indicated sway velocity differences between the control and FOM groups, across both eyes-open and eyes-closed conditions (Table 2). In the eyes-open condition, the FOM group exhibited significantly faster sway velocities in the ML direction compared to the control group in Trial 2 (*t* = −3.95, *p* = 0.001) and Trial 3 (*t* = −2.09, *p* = 0.02). The AP direction showed a significant difference in Trial 2 (*t* = −1.96, *p* = 0.03), but not in other trials. In the eyes-closed condition, the FOM group demonstrated faster ML sway velocity in all trials compared to the control group, with significant differences in Trial 1 (*t* = −2.31, *p* = 0.01), Trial 2 (*t* = −3.09, *p* = 0.001), and Trial 3 (*t* = −2.04, *p* = 0.02). However, AP sway velocity differences were not significant across trials.

**Figure 2:**
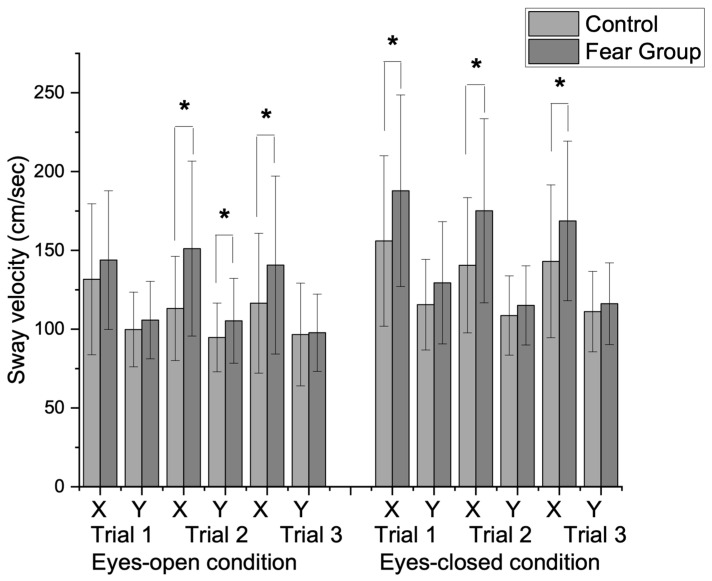
Group differences in sway velocity changes between eyes-open and eyes-closed conditions during repeated one-leg standing trials. A significant group interaction was observed for visual condition (F = 7.43, *p* = 0.01). In the eyes-open condition, there were significant differences in sway velocity in the ML direction during the second trial (*t* = −3.95, *p* = 0.01) and the third trial (*t* = −2.09, *p* = 0.02). The AP direction also showed significant differences during the second trial (*t* = −1.96, *p* = 0.03). In the eyes-closed condition, the fear group demonstrated significantly higher sway velocities in the AP direction across all trials during the first trial (*t* = −2.31, *p* = 0.01), second trial (*t* = −3.09, *p* = 0.001), and third trial (*t* = −2.05, *p* = 0.02). (X: mediolateral direction (ML), Y: anteroposterior direction (AP), * *p* < 0.05).

The mixed repeated-measures ANOVA results indicated the results of sway velocity changes in main effects for Direction (F = 78.59, *p* < 0.001, η^2^p = 0.56), Trial (F = 9.76, *p* = 0.003, η^2^p = 0.14), and Vision (F = 108.95, *p* = 0.001, η^2^p = 0.64). In addition, a significant interaction effect was found between Direction × Vision (F = 27.41, *p* < 0.001, η²p = 0.31), indicating that visual conditions influenced sway velocity differently across AP and ML directions as well as across repeated trials. As shown in Figure 2, there was a significant group interaction on visual condition (F = 7.43, *p* < 0.001, η^2^p = 0.11), which suggested that visual input had a differential effect on sway velocity between groups. These findings indicate that individuals in the FOM group experience faster sway, particularly under eyes-closed conditions in the AP direction.

As shown in Table 3, the correlation analysis revealed distinct relationships between the TSK, age, BMI, and 8 domains of QOL measures between groups with and without FOM. In the control group, TSK negatively correlated with GH (r = −0.41, *p* < 0.05), indicating that higher kinesiophobia was associated with poorer GH perceptions. Age showed significant negative correlations with PF (r = −0.36, *p* < 0.05) and SF (r = −0.45, *p* < 0.05), reflecting declines in these domains with increasing age. No significant relationships were observed between BMI and QOL measures except GH (r = −0.36, *p* < 0.05). The positive inter-domain correlations included RE and SF (r = 0.69, *p* < 0.001), suggesting that improvements in one domain may benefit others.

In the fear group, the TSK showed strong negative correlations with RP (r = −0.55, *p* < 0.001), RE (r = −0.58, *p* < 0.001), EM (r = −0.34, *p* < 0.05), SF (r = −0.69, *p* < 0.001), BP (r = −0.39, *p* < 0.05), and GH (r = −0.32, *p* < 0.05). These results indicate that FOM significantly impairs QOL, particularly affecting social engagement and role limitations due to emotional and physical health issues. Unlike the control group, age did not significantly correlate with QOL domains, and BMI showed no association with QOL. Strong inter-domain correlations were observed, notably between RE and SF (r = 0.76, *p* < 0.001) and between EF and GH (r = 0.66, *p* < 0.001), underscoring the close relationship between emotional well-being and general health in this group.

## 4. Discussion

Our study investigated the impact of FOM on postural control and QOL in older adults under varying visual conditions during one-leg standing trials. The findings demonstrated that visual conditions play a pivotal role in influencing postural stability in older adults with FOM, as increased sway velocities showed their reliance on visual feedback to maintain balance.

We hypothesized that older adults with FOM would exhibit increased sway velocities in the eyes-closed condition and lower QOL measures, particularly in physical functioning. In addition, the QOL scores would be different between groups with and without FOM. This hypothesis was partially accepted, as the FOM group demonstrated significantly faster ML sway velocities under eyes-closed conditions, reinforcing the impact of fear-avoidance behaviors on balance control. The sway velocity analysis revealed that a significant Vision × Group interaction underscores the critical role of visual input in postural control for individuals with FOM. The FOM group’s increased sway velocity in the absence of vision highlights their greater dependence on visual input and potential sensory reweighting deficits. In addition, significant ML sway differences were found in the second and third trials in the eyes-open condition. Their reliance on vision could hinder the development of automatic postural control mechanisms to improve adaptability in dynamic balance tasks. These findings emphasize the need for targeted strategies that progressively reduce visual reliance, enhance proprioceptive and vestibular function, and mitigate fear-avoidance behaviors to improve overall balance and mobility.

In defining kinesiophobia, there is no universally agreed-upon cutoff score on the TSK, as thresholds vary across studies and clinical contexts. Previous research has identified a TSK cutoff of 37/68 (54%) for FOM in older adults with chronic pain [54,55] and 39.39/68 (57%) in another study [4]. However, inconsistent cutoff values may lead to misclassification and limit the generalizability of findings. To enhance the accuracy of identifying FOM, our study aimed to establish a more standardized classification method. In alignment with this approach, we defined the FOM group using a median TSK score threshold of 45% across all subjects, ensuring consistency in participant classification while accounting for individual variations in fear-avoidance behavior.

In the FOM group, TSK showed strong negative correlations with role limitations due to RP and RE, EM, SF, BP, and GH. These findings suggest that FOM substantially impacts social engagement and role limitations associated with both emotional and physical health. Previous studies supported that the kinesiophobia group demonstrated shorter latency times to protect against potential fall risks from perturbations [4,6]. While the FOM group may struggle with integrating sensory information during one-leg standing, they often compensate through somatosensory reweighting strategies to mitigate dynamic balance deficits [5,6,7]. This reliance on compensatory mechanisms underscores the critical role of visual input in the FOM group, as effective postural control requires the integration of visual, vestibular, and proprioceptive inputs to maintain stability [3]. These findings support the hypothesis that fear-induced changes in balance control lead to altered postural dynamics in adults with high levels of kinesiophobia, potentially increasing their risk of falls [28]. Although previous studies have not directly examined the impact of altered proprioception on postural stability, individuals with FOM have been shown to exhibit reduced postural integrity associated with proprioceptive deficits [56]. Given that proprioception is a primary sensory input for postural control, particularly in detecting body sway during static upright standing, its impairment may further exacerbate balance deficits in those with FOM. Investigating the effects of kinesiophobia in older adults is particularly relevant, as these findings highlight the significant influence of psychological factors, such as fear and confidence in balance, on postural control and fall prevention.

Our sway velocity results further underscore the importance of visual feedback in postural control. The significant effects of direction and visual condition demonstrate that sway velocities differ based on movement direction and whether visual feedback is available. Faster sway velocities in the FOM group, particularly under eyes-closed conditions and in the ML direction, highlight the group’s reliance on visual input for balance. These findings support previous work showing that individuals with balance impairments or fear rely heavily on visual cues for stability [15,57,58]. Their studies supported our results to explore effective interventions for improving postural stability in visually impaired individuals and preventing spinal deformity. The interaction between direction and vision further suggests that visual conditions have a differential impact on AP and ML stability. The greater instability observed in the ML direction under eyes-closed conditions may reflect heightened difficulty in compensating for visual deprivation in this plane, particularly in the FOM group. A significant group interaction on visual condition highlighted the critical role of visual input in postural control, while visual conditions significantly modulate ML sways in the FOM group to repeated trials.

The TSK scale was used to assess FOM, or the level of kinesiophobia, distinguishing between non-excessive fear and phobia in patients with musculoskeletal pain. However, there is no universally accepted cutoff score for defining FOM, as thresholds vary across studies and clinical settings. Previous research has identified a TSK cutoff of 37/68 (54%) for FOM in older adults with chronic pain [54,55] and 39.39/68 (57%) in another study [4]. However, inconsistent cutoff values may lead to misclassification and limit the generalizability of findings. To enhance the accuracy of identifying FOM, our study aimed to establish a more standardized classification method. In alignment with this approach, we defined the FOM group using a median TSK score threshold of 45% across all subjects, ensuring consistency in participant classification while accounting for individual variations in fear-avoidance behavior. Although the FOM group in our study exhibited higher TSK scores, these were significantly associated with lower SF-36 scores across most domains except for energy/fatigue. These findings further highlight the substantial impact of kinesiophobia on QOL. Specifically, in the control group, TSK scores were negatively correlated with GH, whereas, in the FOM group, TSK scores showed strong negative correlations with RE, SF, and BP, emphasizing the broader psychological and functional consequences of kinesiophobia.

The QOL outcomes, assessed using the SF-36, revealed significant group differences in all domains except energy/fatigue, suggesting that FOM profoundly affects physical and psychological well-being. Individuals at high risk of falls often exhibit deficits in vestibular function and challenges in integrating sensory information under demanding conditions, which have been identified as critical contributors to balance impairments [4]. These findings further underscore the interconnected roles of sensory systems and visual conditions in maintaining postural stability in older adults. These findings align with prior studies that show that fear-avoidance behaviors can exacerbate functional impairments and limit participation in daily activities [24,25].

Our findings emphasize the sensory reliance and compensatory strategies exhibited by older adults with FOM to maintain postural stability under challenging visual conditions. These results align with previous research indicating that balance declines with age in individuals with visual impairments, necessitating greater reliance on proprioceptive and vestibular systems to compensate for visual deficits [59,60]. Our findings also revealed that visual conditions play a critical role in influencing postural stability among older adults with FOM. Increased sway velocities observed in this group underscore their reliance on visual feedback to maintain balance. These findings suggest that FOM impairs postural control, especially when visual feedback is limited, underscoring the importance of targeted interventions to address fear and improve balance. The higher sway velocities in the fear group under eyes-closed conditions suggest that individuals with FOM may rely heavily on visual input to maintain postural stability, especially without visual cues, and their instability might be exacerbated. Therefore, clinicians may consider targeted balance training to focus on enhancing sensorimotor integration and reducing reliance on visual feedback to improve stability when visual input is unavailable.

Individuals at high risk of falls possess deficits in vestibular function and the ability to integrate sensory information under challenging conditions, which have been identified as critical contributors to balance impairment [4]. While age was negatively correlated with physical functioning and social functioning in the control group, it was not significantly associated with QOL domains in the FOM group. This may reflect the overriding influence of psychological factors, such as fear and avoidance behaviors, on QOL in individuals with FOM. The lack of significant relationships between BMI and QOL measures in both groups suggests that kinesiophobia and sensory reliance play more critical roles in influencing QOL outcomes.

These findings highlight the critical role of visual feedback in maintaining postural stability in older adults with FOM. As a result, rehabilitation programs targeting this population should focus on reducing reliance on visual input by enhancing proprioceptive and vestibular integration. The heightened sway velocities observed under eyes-closed conditions, particularly in the ML direction, indicate a reliance on visual input to maintain balance. This dependence highlights the need for targeted interventions to enhance proprioceptive and vestibular reliance when visual input is unavailable. Our findings provide valuable insights into rehabilitation strategies focusing on anticipatory postural adjustments, which should integrate separate mechanisms for visual, proprioceptive, and vestibular inputs to improve balance and postural stability. Addressing these isolated components in clinical practice will enhance balance training, reduce fall risk, and improve QOL for older adults with postural deficits. In addition, the observed correlations between TSK and QOL domains underscore the need for holistic interventions that address both psychological and physical factors.

Significant differences in ML sway across trials under eyes-closed conditions were observed, emphasizing the relevance of lateral-plane motion in postural stability. Previous research has indicated that ML sway is a strong predictor of fall risk in older adults [61]. Furthermore, difficulties in controlling ML postural stability are closely tied to fear of falling, which influences static postural control [62]. Our findings align with studies that suggest that ML sway deficits are a critical aspect of postural instability in older adults with FOM. The increased postural instability in the ML direction observed in this study underscores the need for tailored interventions to improve ML sway control, particularly under eyes-closed conditions. These findings highlight that individuals with high fall efficacy may benefit from repeated trials to reduce ML sway deficits [63]. The use of repeated standing trials in our study protocol provides valuable insights into adaptive strategies that minimize fall risk in the ML direction under eyes-closed conditions. However, previous studies have largely focused on younger older adults, introducing potential confounding effects. Our study emphasizes the importance of addressing ML sway in older adults to mitigate fall risk and improve postural control.

Our findings align with previous studies examining proprioceptive reweighting mechanisms in response to disturbances in signals from the paraspinal and calf muscles during standing [4,17,64]. These studies highlight how the central nervous system adjusts the relative weighting of proprioceptive inputs to maintain standing balance, a critical adaptive mechanism for individuals with FOM. Our results also indicated that reliance on an ML strategy highlights the clinical importance of repeated trials in understanding balance deficits and motor control strategies. Prior research suggests that individuals with FOM may adopt a “tight control strategy”, characterized by reduced spinal segment mobility and co-contraction of trunk and lower-limb muscles, to enhance kinematic and kinetic stability [65,66]. This strategy may represent a pain-avoidance mechanism to minimize fall risk in the FOM group.

In general, greater postural sway has been linked to an increased risk of falling in older adults [67]. However, the systematic reviews have not reported a consistent correlation between pain intensity and the magnitude of COP excursions, likely due to variability in study designs and confounding factors [68,69]. These inconsistencies highlight the need for comprehensive assessments of balance and pain characteristics to elucidate mechanisms underlying the relationship between pain, instability, and fear of falling [70,71]. Our findings are consistent with previous research indicating that sagittal imbalance in older adults is associated with diminished trunk proprioceptive input and postural instability [72]. Poor momentum control, as an underlying mechanism of postural imbalance and falls, warrants further investigation in rehabilitation contexts [73].

The results of our study contribute to the clinical understanding of neuromuscular control, particularly in ML sway velocities under eyes-closed conditions. Optimizing ML sway control is crucial for effective postural training. Anticipatory postural adjustments, which involve coordinated muscle activation to minimize sway velocities during dynamic stability tasks, such as one-leg standing, play a key role in maintaining balance [74,75]. The compensatory movement patterns observed in this population likely aim to reduce fear and enhance confidence during dynamic standing balance tasks. Adjustments in sway velocities appear designed to prevent fall injuries. If sensorimotor control mechanisms predominantly govern balance across varying visual conditions, one might expect similar sway velocities between eyes-open and eyes-closed conditions during one-leg standing tasks. However, the differences in sway observed in this study may reflect the novelty of the experience and coordination challenges, as neuromuscular reconnection is required to compensate for musculoskeletal pain and dysfunction.

Our study has several limitations. First, its cross-sectional design prevents causal inferences and the broad age range among older adults. In addition, the FOM group was classified based on the median TSK score, categorizing participants into high and low TSK groups rather than using clinically validated cutoffs. This classification method may not fully reflect the clinical relevance of kinesiophobia, potentially affecting the accuracy of participant categorization and limiting the generalizability of our findings. Second, physiological factors, such as fatigue accumulation, trial duration, and anthropometric differences, may have influenced sway velocity outcomes. Third, despite conducting preliminary power analyses, the relatively small sample size remains a limitation, reducing the generalizability of our findings.

Future research should utilize larger cohorts and longitudinal designs to validate these results and further investigate the mechanisms underlying postural control differences. Such studies would provide a more comprehensive understanding of the relationship between FOM, postural strategies, and QOL, ultimately guiding targeted interventions to improve functional outcomes. Fear-avoidance behaviors may further contribute to postural adjustments, emphasizing the importance of integrating vision-based strategies and confidence in movement.

## 5. Conclusions

The FOM group demonstrated a heightened reliance on vision, underscoring the need for vision-dependent balance strategies. Fear-avoidance behaviors may contribute to altered postural adaptations, reinforcing the importance of integrating vision-based strategies and progressive balance training. In addition, the strong correlations between TSK scores and QOL domains highlight the necessity of holistic interventions that address both psychological and physical factors.

## Figures and Tables

**Table 1:** Summary of group differences in subject anthropometric variables and QOL measures.

Variables	Control Group (*n* = 37)	Fear Group (*n* = 36)	Statistics	*p*	95% CI (Upper/Lower)
Age (years)	73.27 ± 8.19	72.86 ± 6.92	*t* = 0.23	0.41	−3.13/3.95
BMI (kg/m^2^)	27.21 ± 0.04	27.90 ± 0.05	*t* = −0.59	0.27	−0.03/0.16
TSK	33.65 ± 0.07	58.02 ± 10.36	*t* = −11.78	0.001 **	−0.28/−0.20
SF36-PF	73.59 ± 27.51	59.25 ± 26.22	*t* = 2.27	0.01 *	1.78/26.88
SF36-RP	80.01 ± 35.89	51.88 ± 41.36	*t* = 3.24	0.001 **	10.89/45.36
SF36-RE	89.74 ± 30.75	70.01 ± 39.79	*t* = 2.46	0.01 *	3.77/35.69
SF36-EF	65.40 ± 14.73	51.74 ± 20.44	*t* = 3.28	0.001 **	5.37/21.96
SF36-EM	79.05 ± 16.06	72.92 ± 16.79	*t* = 1.59	0.06	−1.53/13.81
SF36-SF	93.91 ± 14.31	78.12 ± 24.16	*t* = 3.41	0.001 **	6.55/25.03
SF36-BP	79.53 ± 20.94	60.97 ± 21.69	*t* = 3.72	0.001 **	8.60/28.51
SF36-GH	75.74 ± 12.51	59.79 ± 19.005	*t* = 4.24	0.001 **	8.44/23.45

QOL: quality of life; BMI: body mass index; TSK: Tampa Scale of Kinesiophobia; SF36: short-form 36-item questionnaire; CI: confidence interval; PF: physical functioning; RP: role limitations due to physical health; RE: role limitation due to emotional problems; EF: energy/fatigue; EM: emotional well-being; SF: social functioning; BP: bodily pain; GH: general health. * *p* < 0.05, ** *p* < 0.01.

**Table 2:** Summary of group differences in sway velocity measures considering visual conditions.

Variables	Control Group (*n* = 37)	Fear Group (*n* = 36)	Statistics	*p*	95% CI (Upper/Lower)
EO T1 X	131.69 ± 47.04	145.01 ± 50.82	*t* = −1.14	0.12	−36.49/9.84
EO T1 Y	99.16 ± 23.21	105.59 ± 24.62	*t* = −1.13	0.13	−17.57/4.99
EO T2 X	112.12 ± 33.19	156.36 ± 57.14	*t* = −3.95	0.001 **	−66.56/−21.91
EO T2 Y	94.24 ± 21.74	105.60 ± 25.95	*t* = −1.96	0.03 *	−22.90/0.20
EO T3 X	117.69 ± 44.32	144.64 ± 59.37	*t* = −2.09	0.02 *	−52.66/−1.24
EO T3 Y	97.31 ± 32.44	98.70 ± 24.63	*t* = −0.19	0.42	−15.97/13.17
EC T1 X	153.46 ± 53.66	185.63 ± 63.70	*t* = −2.31	0.01 **	−59.97/−4.35
EC T1 Y	113.94 ± 28.71	125.81 ± 37.26	*t* = −1.51	0.07	−27.55/3.79
EC T2 X	138.26 ± 44.49	178.42 ± 62.15	*t* = −3.09	0.001 **	−66.12/−14.20
EC T2 Y	107.97 ± 25.17	114.44 ± 25.06	*t* = −1.04	0.15	−18.88/5.96
EC T3 X	143.04 ± 48.47	166.65 ± 50.62	*t* = −2.04	0.02 *	−50.69/−0.56
EC T3 Y	111.13 ± 25.51	116.17 ± 25.93	*t* = −0.77	0.22	−18.07/7.98

EO: eyes-open condition; EC: eyes-closed condition; T: one-leg standing trial; X: mediolateral direction; Y: anteroposterior direction. * *p* < 0.05, ** *p* < 0.01.

**Table 3:** Correlation analysis results between TSK, age, BMI, and QOL measures by SF-36.

Control Group	TSK	Age	BMI	PF	RP	RE	EF	EM	SF	BP	GH
TSK											
Age	−0.31										
BMI	0.24	−0.32									
PF	−0.19	−0.36 *	0.03								
RP	−0.35 *	−0.32 *	−0.10	0.33 *							
RE	−0.27	−0.29	−0.16	0.38 **	0.65 **						
EF	−0.21	−0.07	−0.23	0.33 *	0.69 **	0.46 *					
EM	0.12	0.11	0.28	0.29	0.13	0.26	0.23				
SF	−0.31	−0.45 *	0.11	0.31	0.67 **	0.69 **	0.47 *	0.22			
BP	−0.14	−0.05	0.07	0.13	0.48 **	0.45 *	0.39 *	0.17	0.47 *		
GH	−0.41 *	−0.09 *	−0.36 **	0.07	0.55 **	0.39 *	0.46 *	0.04	0.36 *	0.15	
Fear Group	TSK	Age	BMI	PF	RP	RE	EF	EM	SF	BP	GH
TSK											
Age	0.13										
BMI	−0.07	−0.28									
PF	−0.28	0.01	−0.18								
RP	−0.55 **	−0.03	−0.15	0.53 **							
RE	−0.58 **	−0.08	−0.19	0.48 **	0.51 **						
EF	−0.19	−0.09	−0.01	0.49 **	0.49 *	0.27					
EM	−0.34 *	0.22	0.14	0.41 **	0.39 *	0.25	0.64 **				
SF	−0.69 **	0.14	−0.04	0.36 *	0.41 *	0.76 **	0.41 *	0.51 *			
BP	−0.39 *	0.12	0.26	0.42 *	0.46 *	0.54 **	0.38 *	0.27	0.43 *		
GH	−0.32 *	0.03	−0.03	0.54 **	0.58 **	0.36 *	0.66 **	0.47 *	0.41 *	0.48 *	

TSK: Tampa Scale of Kinesiophobia; BMI: body mass index; SF-36: short-form 36-item questionnaire; PF: physical functioning; RP: role limitation due to physical health; RE: role limitation due to emotional problems; EF: energy/fatigue; EM: emotional well-being; SF: social functioning; BP: bodily pain; GH: general health. * *p* < 0.05, ** *p* < 0.001.

## Data Availability

The data that supported the findings of this study are available from the corresponding author upon request.

## Abbreviations

The following abbreviations are used in this manuscript:

FOM	fear of movement
QOL	quality of life
TSK	Tampa Scale of Kinesiophobia
SF36	short-form 36-item questionnaire
PF	physical functioning
RP	role limitations due to physical health
RE	role limitation due to emotional problems
EF	energy/fatigue
EM	emotional well-being
SF	social functioning
BP	bodily pain
GH	general health
EO	eyes-open condition
EC	eyes-closed condition
T	one-leg standing trial
X	mediolateral direction
Y	anteroposterior direction
ML	mediolateral
